# Complement Anaphylatoxins and Inflammatory Cytokines as Prognostic Markers for COVID-19 Severity and In-Hospital Mortality

**DOI:** 10.3389/fimmu.2021.668725

**Published:** 2021-07-01

**Authors:** Bandar Alosaimi, Ayman Mubarak, Maaweya E. Hamed, Abdullah Z. Almutairi, Ahmed A. Alrashed, Abdullah AlJuryyan, Mushira Enani, Faris Q. Alenzi, Wael Alturaiki

**Affiliations:** ^1^ Research Center, King Fahad Medical City, Riyadh, Saudi Arabia; ^2^ College of Medicine, King Fahad Medical City, Riyadh, Saudi Arabia; ^3^ Department of Botany and Microbiology, College of Science, King Saud University, Riyadh, Saudi Arabia; ^4^ Laboratory and Blood Bank, King Fahad Hospital, Madina, Saudi Arabia; ^5^ Pharmaceutical Service Department, Main Hospital, King Fahad Medical City, Riyadh, Saudi Arabia; ^6^ Pathology and Clinical Laboratory Management, King Fahad Medical City, Riyadh, Saudi Arabia; ^7^ Medical Specialties Department, King Fahad Medical City, Riyadh, Saudi Arabia; ^8^ Department of Medical Laboratory Sciences, College of Applied Medical Sciences, Prince Sattam bin Abdulaziz University, Alkharj, Saudi Arabia; ^9^ Department of Medical Laboratory Sciences, College of Applied Medical Sciences, Majmaah University, Majmaah, Saudi Arabia

**Keywords:** COVID-19, SARS‐CoV‐2, complement anaphylatoxins, inflammatory cytokines, in-hospital mortality, prognosis

## Abstract

COVID-19 severity due to innate immunity dysregulation accounts for prolonged hospitalization, critical complications, and mortality. Severe SARS-CoV-2 infections involve the complement pathway activation for cytokine storm development. Nevertheless, the role of complement in COVID-19 immunopathology, complement‐modulating treatment strategies against COVID-19, and the complement and SARS‐CoV‐2 interaction with clinical disease outcomes remain elusive. This study investigated the potential changes in complement signaling, and the associated inflammatory mediators, in mild-to-critical COVID-19 patients and their clinical outcomes. A total of 53 patients infected with SARS-CoV-2 were enrolled in the study (26 critical and 27 mild cases), and additional 18 healthy control patients were also included. Complement proteins and inflammatory cytokines and chemokines were measured in the sera of patients with COVID-19 as well as healthy controls by specific enzyme-linked immunosorbent assay. C3a, C5a, and factor P (properdin), as well as interleukin (IL)-1β, IL-6, IL-8, tumor necrosis factor (TNF)-α, and IgM antibody levels, were higher in critical COVID-19 patients compared to mild COVID-19 patients. Additionally, compared to the mild COVID-19 patients, factor I and C4-BP levels were significantly decreased in the critical COVID-19 patients. Meanwhile, RANTES levels were significantly higher in the mild patients compared to critical patients. Furthermore, the critical COVID-19 intra-group analysis showed significantly higher C5a, C3a, and factor P levels in the critical COVID-19 non-survival group than in the survival group. Additionally, IL-1β, IL-6, and IL-8 were significantly upregulated in the critical COVID-19 non-survival group compared to the survival group. Finally, C5a, C3a, factor P, and serum IL-1β, IL-6, and IL-8 levels positively correlated with critical COVID-19 in-hospital deaths. These findings highlight the potential prognostic utility of the complement system for predicting COVID-19 severity and mortality while suggesting that complement anaphylatoxins and inflammatory cytokines are potential treatment targets against COVID-19.

## Introduction

Sever Acute Respiratory Syndrome Coronavirus 2 (SARS-CoV-2) was reported in China on Dec 31, 2019, after several unexplained cases of pneumonia were reported (1, 2). COVID-19 cases are defined as symptomatic, asymptomatic, and severe disease with mortality rates highest among patients with chronic disease, the elderly, and immunocompromised patients (3). The World Health Organization declared the outbreak a public health emergency of international concern On January 30, 2020 (2). Following community transmission of SARS-CoV-2 in many countries, the WHO declared the COVID-19 outbreak as a pandemic on March 12, 2020. By December 3, 2020, SARS-CoV2 had spread to 206 countries with a total of 63,719,213 laboratory-confirmed cases and 1,482,084 deaths reported around the world (1–4). Severe cases of COVID-19 cannot be predicted early during the onset of symptoms due to the lack of biomarkers or accurate testing for the prediction of COVID-19 disease severity (5, 6). As such, identification of immune parameters as diagnostic biomarkers to predict COVID-19 disease severity might help to accurately select interventional strategies (7–9).

Immune response dysregulation is a primary characteristic of COVID-19 severity, disease evolution and worse clinical outcomes (10). Accumulated evidence suggests that SARS-CoV-2 infections are characterized by dysregulation of innate and adaptive immunity (10–12). Several inflammatory cytokines and chemokines (interleukin (IL)-1β, IL-2, IL-6, IL-7, IL-8, IL-10, granulocyte colony-stimulating factor (G-CSF), granulocyte/macrophage colony-stimulating factor (GM-CSF), interferon gamma-induced protein (IP)-10, monocyte chemoattractant protein (MCP)-1, macrophage inflammatory protein (MIP)-1α, interferon (IFN)-γ, tumor necrosis factor (TNF)-α, C-C motif chemokine ligand (CCL)2 and CCL3) as well as C-reactive protein (CRP) are significantly upregulated in severe and critical COVID-19 patients (13, 14). The high inflammatory cytokines and chemokine levels during SARS‐CoV-1, MERS‐CoV, and SARS‐CoV‐2 infections are strongly associated with massive infiltration of immune cells into the lungs and poor disease outcomes (15, 16).

The complement system comprises a proteins network (17). Depending on the type of activation factors, the complement cascade is activated through three pathways: The classical pathway, lectin pathway, and alternative pathway (18–20). Crosstalk between the complement and coagulation systems has been reported to play a crucial role in the vascular endothelial damage and thromboinflammation (21). The complement system is negatively regulated by various complement proteins, including factor I, C1-inhibitor (CI-INH), factor H and C4-binding protein (C4-BP) (11, 21); meanwhile, factor P (properdin) is a positive regulatory complement protein. Several viral infections are associated with complement activation and coagulation dysfunctions (22). Overactivation of pulmonary and systemic complement plays a key role in inflammation, endothelial cell damage, thrombus formation, intravascular coagulation, and multiple organ failure, ultimately leading to death (23, 24). C5a is chemoattractant for neutrophils, monocytes, eosinophils, and T lymphocytes (24). Following infection, complement anaphylatoxins stimulate macrophages to produce TNF-α, IL‐1β, IL‐6, and IL‐8; these mediators promote vascular dysfunction, fibrinolysis, and microvascular thrombosis formation (24). The C5a also plays a major role in inducing higher expression of P‐selectin, intercellular adhesion molecule‐1, fibronectin and fibrinogen. This upregulation of adhesion molecules provokes various cell signaling and pro‐inflammatory pathways (25).

The role of complement in COVID-19 immunopathology and complement‐modulating treatment strategies against COVID-19 has received limited attention with several unanswered questions regarding the interaction between complement, SARS‐CoV‐2, and clinical disease outcomes. In this study, we comprehensively investigated the complement system, pro-inflammatory cytokines/chemokines, and antibody responses of patients with critical and mild COVID-19. The correlation between all examined parameters and COVID-19 in-hospital mortality was also assessed. To the best of our knowledge, this is the first study to investigate the functionality of several complement proteins and complement regulatory factors in the context of critical COVID-19 cases.

## Materials and Methods

### Patient Selection, Setting, and Sample Collection

This study enrolled 53 patients infected with SARS-CoV-2, as confirmed by RT‐PCR (inclusion criterion). There were 26 critical (intensive care unit [ICU]) and 27 mild COVID-19 cases, and 18 healthy matched control groups. The mild cases were defined based on the absence of symptoms such as shortness of breath and pneumonia and other less severe clinical symptoms (low-grade fever and cough). Blood samples were collected, allowed to clot for 20–35 min at 25°C and subjected to centrifugation for 15 min at 1,000 × *g*. The serum was immediately assayed, liquated, and stored at ≥ −20°C. Repeated freeze-thaw cycles were avoided. The exclusion criteria were: 1) patients under 14 years of age; 2) patients co-infected with other respiratory pathogens; 3) patients diagnosed with bacteremia and/or viremia caused by any other viruses; 4) immunocompromised patients; 5) patients under treatment with anti-inflammatory and/or anti-complement drugs; 6) patients with preexisting autoimmune diseases; 7) women who were either pregnant or lactating (**Table 1**). This study was reviewed and approved by the Institutional Review Board of King Fahad Medical City (IRB register number 20‒193) and written informed consent was obtained from all subjects prior to enrollment.

**Table 1:** Demographics and clinical characteristics of COVID-19 patients.

Baseline variables	All patients	Mild	Critical	*p*-value
	n = 53	n = 27 (51%)	n = 26 (49%)	
**Demographics**				
**Age**				
Median	55 ± 18	46 ± 19	63 ± 12	
Range	16–92	16–92	25–82	
**Gender**				
Male	41 (77%)	21 (78%)	20 (77%)	0.94
Female	12 (23%)	6 (22%)	6 (23%)	
**Ethnicity**				
Saudi	19 (36%)	6 (22%)	13 (50%)	0.03
Non-Saudi	34 (64%)	21 (78%)	13 (50%)	
**Case Fatality Rate**	11 (21%)	–	11 (100%)	

### Clinical Laboratory Investigations

The following blood parameters were examined: CRP, INR, PT, PTT, creatine kinase, Creatine Kinase Myocardial Band, Trop, Hb, platelet, red blood cells, WBC, glucose, ESR, low-density lipoprotein cholesterol, AST, ALT, urea, creatinine, high-density lipoprotein cholesterol, albumin, and total protein.

### Measurement of Complement Inflammatory Mediators Anaphylatoxins (C3a/C3b and C5a) and Complement Classical Pathway Component (C1q) and C2 Levels

Serum levels of human complement C3 and C5 fragments, as well as C3b, C1q, and C2 were measured according to the manufacturer’s guidelines using enzyme-linked immunosorbent assay (ELISA) kits (HCA39-K01-Eagle Biosciences, Inc., Columbia, USA; ab193695, Abcam, Cambridge, UK; ab195461, Abcam; ab170246, Abcam; ab154132, Abcam). The concentrations were calculated using standard curves.

### Quantification of Serum Complement Regulatory Components (Factors) Levels

Serum concentrations of four complement regulatory factors, P, I, C4-BP, and H were quantified using ELISA kits (ab222864, Abcam; ab195460, Abcam; ab222866, Abcam; HK342, Hycult Biotech, Uden, Netherlands). All ELISA protocols were performed according to the manufacturer’s instructions. The concentrations were calculated using standard curves.

### Quantification of Serum Chemokine RANTES (CCL5) Levels

Serum RANTES levels were quantified in COVID-19 infection patients (n = 53) and healthy volunteers (n = 18) using a human RANTES ELISA kit (R&D Systems, Minneapolis, USA) following the manufacturer’s protocol. The concentrations were calculated using standard curves, and the results were expressed as pg/mL.

### Quantification of the SARS-CoV-2 Antibody

The SARS-CoV-2 IgM antibody was quantified using ELISA kits (AnshLabs, Webster, USA). The SARS-CoV-2 IgM ELISA assay detects antibodies against the spike and nucleocapsid proteins. The assay was performed according to the manufacturer’s protocol. IgM concentration > 12 AU/mL (positive cutoff) was considered positive.

### Quantification of Pro-Inflammatory Cytokine and Chemokine Profiles Using ELISArray

The concentrations of the primary 12 human pro-inflammatory cytokines and chemokines (IL1-α, IL1-β, IL-2, IL-4, IL-6, IL-8, IL-10, IL-12, IL-17A, IFN-γ, TNF-α, and GM-CSF) were measured in the serum of 53 COVID-19 patients and healthy controls, using the multi-analyte ELISArray (Qiagen, Germantown, MD, USA) following the manufacturer’s protocol. The absorbance of the ELISArray was measured at 450 nm and concentrations were calculated using a standard curve. The cytokine or chemokine levels were expressed as pg/mL.

### Statistical Analysis

All statistical analyses were performed using GraphPad 5.0 (GraphPad Software, San Diego, CA, USA). Data were assessed using one-way ANOVA followed by Tukey’s multiple comparison test. The correlations between complement proteins, inflammatory cytokines/chemokines, and clinical laboratory blood parameters were tested using the Pearson correlation. Results are presented as mean  ± standard deviation unless otherwise specified. A *p*-value of < 0.05 was considered statistically significant.

## Results

### Basic Characteristics of COVID-19 Patients

A total of 53 COVID-19 patients (26 critical and 27 mild) were included in this study (41 males and 12 females), with an age ranging from 18 to 92 years and a mean age of 53.2 ± 18.0 years. The mean age was 61.6 ± 11.8 years in the critical group, 45.2 ± 19.2 years in the mild group, and 66.2 ± 6.5 and 58.3 ± 13.6 years in the non-survival and survival patients, respectively. Eleven (42.3%) patients died while 15 recovered (57.7%) in the critical COVID-19 group. All basic characteristics of the enrolled patients are shown in **Table 1**.

### Circulating Complement Activation in Critical and Mild COVID-19 Patients

**Figure 1:**
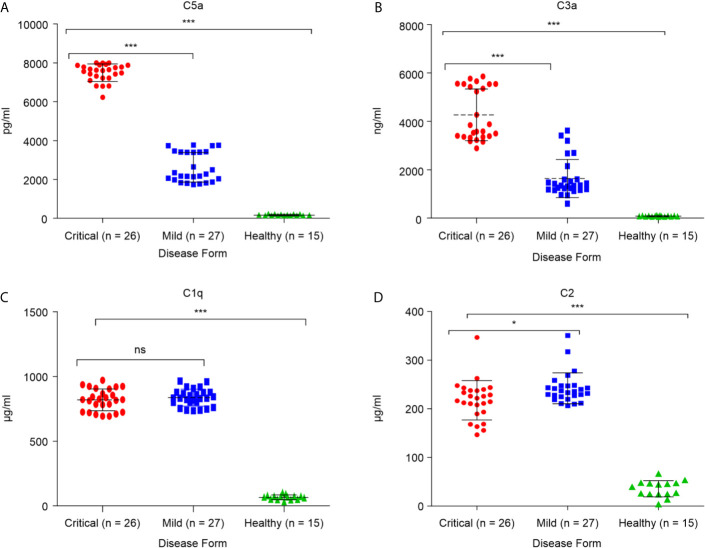
Serum concentrations of the anaphylatoxins in critical and mild COVID-19 patients, and healthy controls. Concentrations of **(A)** complement fragment C5a; **(B)** complement fragment C3a; **(C)** complement fragment C1q; and **(D)** complement fragment C2 were measured using ELISA. ANOVA and Tukey’s multiple comparison test were used for statistical comparisons. *p < 0.05, ***p < 0.001, and ns *p* > 0.05.

To determine whether critical COVID-19 patients have high levels of the complement inflammatory mediators anaphylatoxins C3a and C5a, systemic levels of complement (anaphylatoxins) C3a, a product of C3 cleavage, and C5a, a product of C5 cleavage, were measured in samples from patients with confirmed SARS-CoV-2 infection (26 critical and 27 mild), and 17 healthy groups. As shown in **Figures 1A, B**, both C3a and C5a levels were higher in the COVID group than in the control group. Additionally, severe patients had higher levels of these markers than patients with mild disease (p < 0.0001). The elevated levels of C3a and C5a observed in the critical COVID-19 patients were consistent with decreased levels of complement negative regulatory factor I and C4-BP in the same patients. Overall, these results illustrate the role of complement anaphylatoxins C5a and C3a in pulmonary immunopathology, endothelial damage, vascular leakage, and case fatality in patients with critical COVID-19. The C2 levels were higher in the COVID group than in the control group (**Figure 1D**). Interestingly, C2 was higher in mild patients compared to severe patients, while there were no significant differences in the C1q levels between these two groups (**Figure 1C**).

### Critical COVID-19 Condition Related to Abnormal Levels of Complement Regulatory Proteins

#### Critical COVID-19 Is Associated With Positive Regulation of Complement Activation

**Figure 2:**
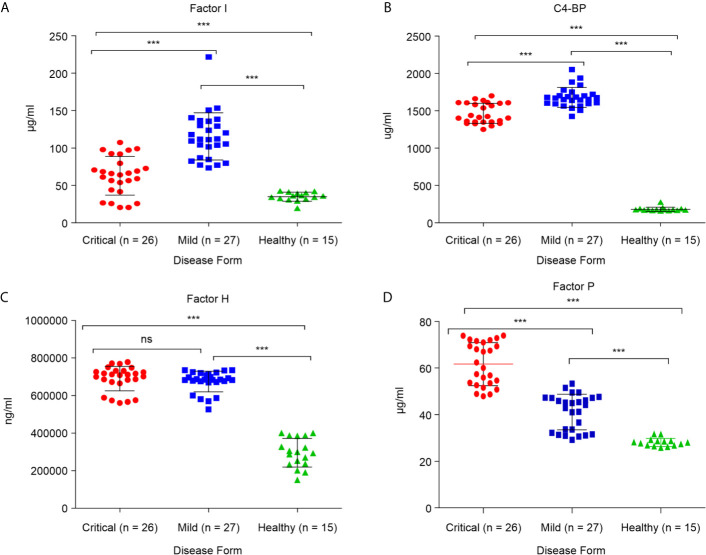
Serum concentrations of complement factors in the critical and mild COVID-19 patients, and healthy controls. Concentrations of **(A)** factor I; **(B)** factor C4-BP; **(C)** factor H; and **(D)** factor P. ANOVA and Tukey’s multiple comparison test were used for statistical comparisons. ***p < 0.001 and ns *p* > 0.05.

These alterations in major complement proteins led us to investigate whether there are changes in regulators of this pathway. The complement regulatory proteins are crucial for controlling complement overactivation to avoid inflammatory pathologies and tissue damage. To determine whether the complement regulatory factors were altered during SARS-CoV-2 infection, four complement regulatory factors, namely factor P, factor I, C4-BP, and factor H, were quantified in critical and mild COVID-19 patients. As shown in **Figure 2D**, the levels of positive regulatory factor P were significantly higher in the critical COVID-19 patients than in the mild COVID-19 and non-COVID-19 healthy control groups (*p* < 0.0001), suggesting that the levels of factor P may positively regulate complement activation during the SARS-CoV-2 infection. Furthermore, measurement of factor P in serum might provide evidence for the involvement of the alternative complement pathway since factor P is an important factor in the alternative pathway activation.

#### Critical COVID-19 Cases Are Associated With Decreased Levels of Complement Negative Regulatory Proteins

Measurement of negative regulatory proteins in COVID-19 patients may provide evidence for complement cascade regulation. Quantitation of the complement negative regulatory factors (factor I, C4-BP, and factor H), in critical and mild COVID-19 patients showed that the levels of negative regulatory proteins, factor I and C4-BP, were significantly lower in the critical COVID-19 group than in the mild COVID-19 group (*p* < 0.0001; **Figures 2A, B**). These negative regulatory proteins play a critical role in complement system regulation. The low levels of factor I and C4-BP suggest that critical COVID-19 patients have a reduced ability to control and regulate complement activation, suggesting that SARS-CoV-2 somehow suppresses and inhibits the complement negative regulatory proteins during critical COVID-19. In contrast, the level of factor H, which regulates complement by inactivating the C3 convertase and dislodges Bb from the C3bBb complex, did not differ between the critical and mild COVID-19 patients (*p* > 0.05; **Figure 2C**).

### Critical COVID-19 Induces High Levels of SARS-CoV-2 IgM Antibody

**Figure 3:**
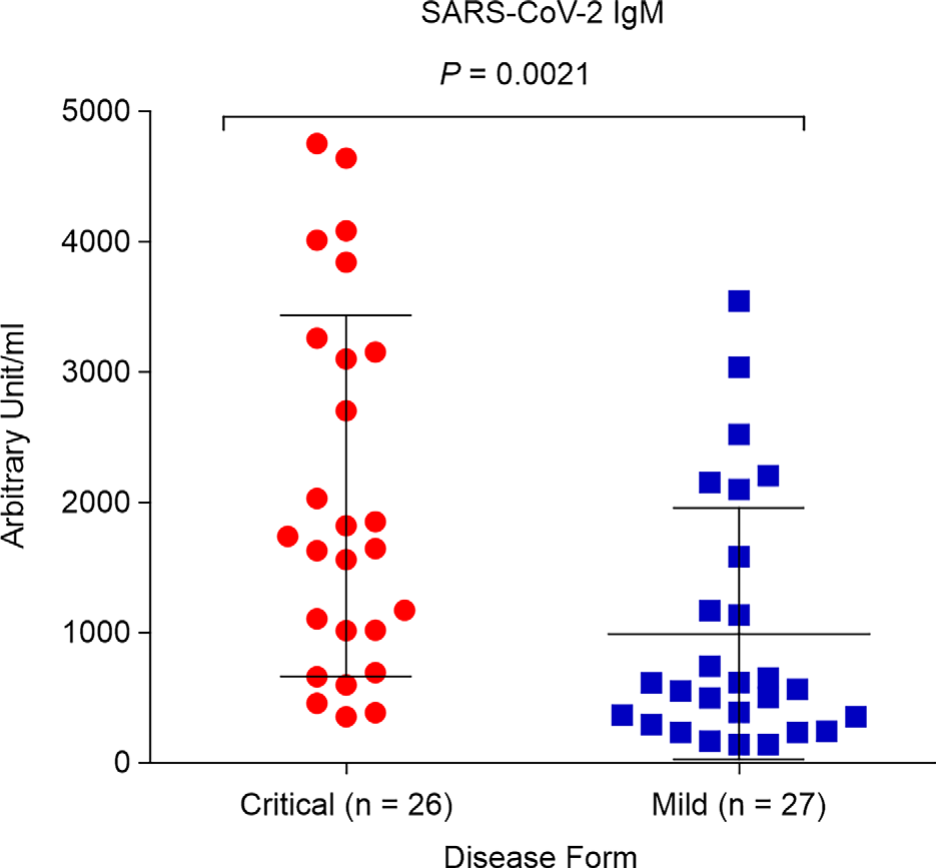
Levels of SARS-CoV-2 immunoglobulin M (IgM) in critical and mild COVID-19 patients (*P* = 0.002). ELISA assay was used to detect antibodies against spike and nucleocapsid protein.

We aimed to determine whether SARS-CoV-2 IgM contributes to COVID-19 severity. Both critical and mild COVID-19 patients showed positive IgM antibody response, with mean values of 2,050.7 AU/mL and 992.2 AU/mL, respectively (positive cutoff > 12 AU/mL). However, the IgM concentration in critical COVID-19 patients was significantly higher than that in patients with mild COVID-19 (*p =* 0.0021; **Figure 3**).

### RANTES (CCL5) Levels Are Increased in Mild COVID-19 Patients

**Figure 4:**
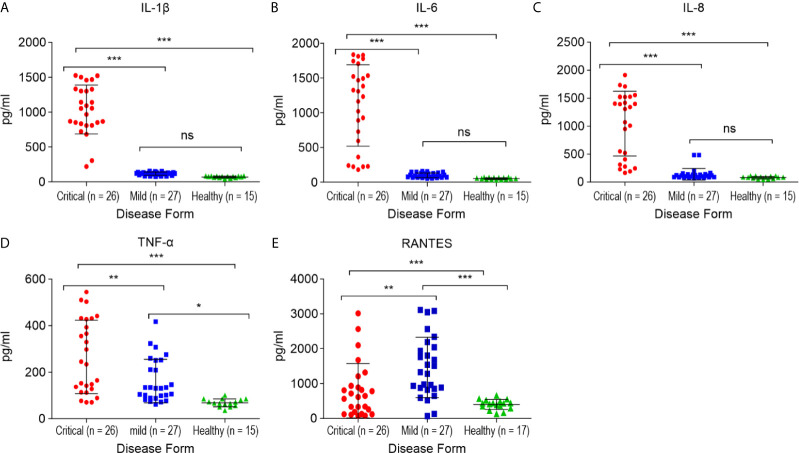
Comparison of the cytokine/chemokine levels between the critical and mild COVID-19 patients, and healthy controls. **(A)** interleukin 1 β (IL-1β); **(B)** interleukin 6 (IL-6); **(C)** interleukin 8 (IL-8); **(D)** tumor necrosis factor α (TNF-α); **(E)** RANTES. *p < 0.05, **p < 0.01 and ***p < 0.001 and ns p > 0.05.

Alterations in the concentration of serum RANTES in the studied patients with mild COVID-19 and non-COVID-19 healthy controls are shown in **Figure 4E**. The serum RANTES levels were higher in the mild COVID-19 group than in the critical COVID-19 and healthy control groups (*p* < 0.01, for both). RANTES during mild SARS-CoV-2 infection might play a role in the anti-SARS-CoV-2 immune response.

### Critical COVID-19 Patients Exhibit High Levels of IL-1β, IL-6, TNF-α, and Neutrophil Chemoattractant Chemokine IL-8 (CXCL8)

The serum samples from critical COVID-19, mild COVID-19, and healthy controls were collected to quantify 12 pro-inflammatory cytokines and chemokines. The levels of IL1-β, IL-6, IL-8 (*p <* 0.0001, for each), and TNF-α (*p* < 0.01) were significantly higher in the critical COVID-19 patients than in the mild COVID-19 and non-COVID-19 healthy controls (**Figure 4**).

### Critical COVID-19 Non-Survival Patients Have Elevated Levels of Complement Components and Pro-Inflammatory Cytokines/Chemokines

**Figure 5:**
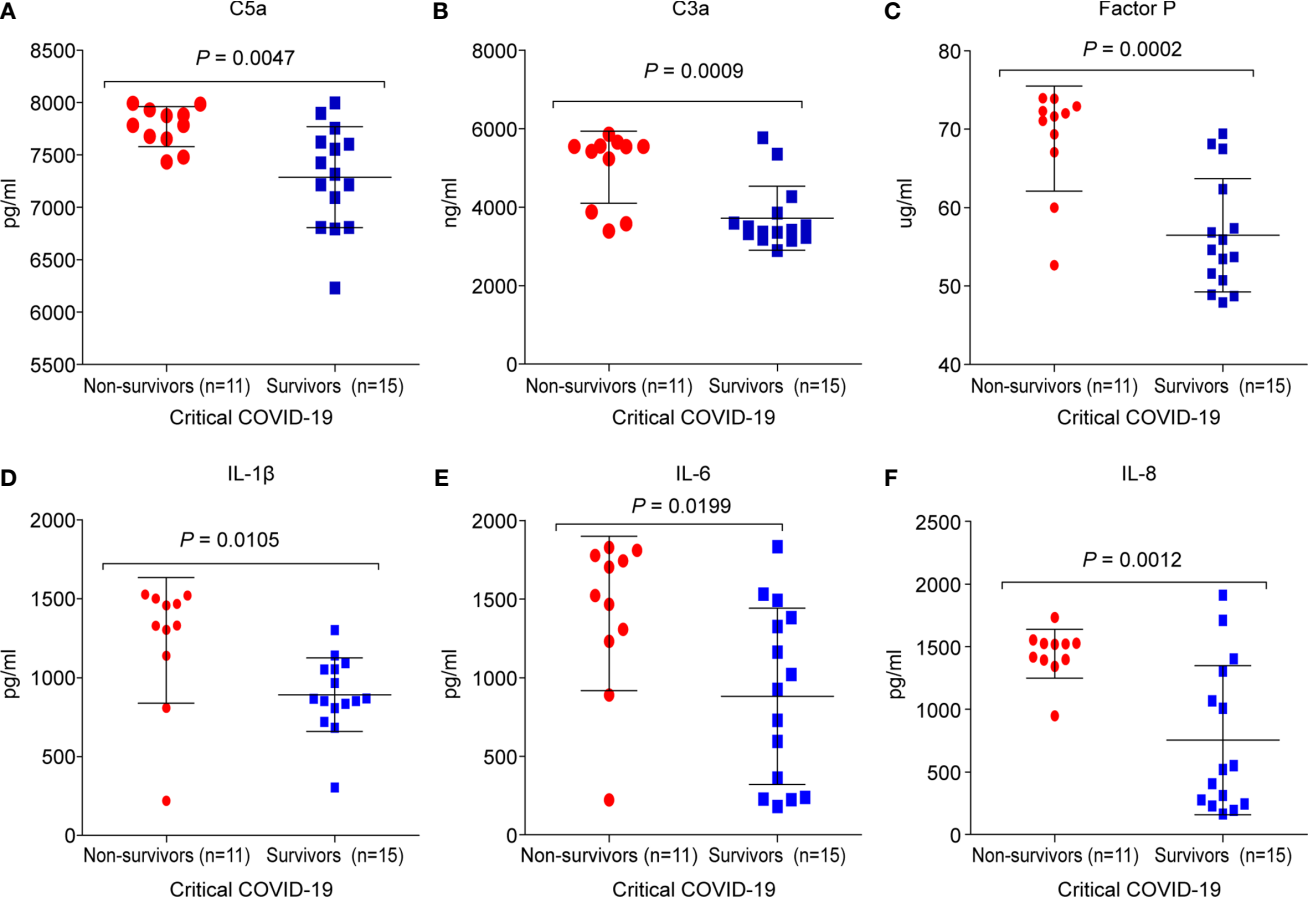
Comparison of the complement proteins and cytokine/chemokine levels between critical COVID-19 patients who survived and those who did not survive. The serum samples were collected from the day of admission or day 14 during the intensive care unit (ICU)stay. **(A)** complement fragment C5a (*P* = 0.005); **(B)** complement fragment C3a (*P* < 0.001); **(C)** factor P (*P* < 0.001); **(D)** interleukin 1 β (IL-1β) (*P* = 0.01); **(E)** interleukin 6 (IL-6) (*P* = 0.02); **(F)** interleukin 8 (IL-8) (*P* = 0.001).

It was found that 11/26 (42%) critical COVID-19 patients died during hospitalization. The C5a (7,770.3 ± 191; p =0.0047), C3a (5,019.2 ± 920.4; p = 0.0008), and factor P (68.8 ± 6.7; p = 0.0002) concentrations were significantly higher in the critical COVID-19 non-survival group than in the critical COVID-19 survival group (**Figure 5**). Additionally, the concentrations of IL-1β (1,236.5 ± 398.1; p = 0.0105), IL-6 (1,408.9 ± 490.3; p = 0.0198), and IL-8 (1,443.4 ± 195.8; p = 0.0012) were significantly upregulated in the critical COVID-19 non-survival group compared to the critical COVID-19 survival group (**Figure 5**).

### Association Between Circulating Levels of Complement Proteins and Pro-Inflammatory Cytokines/Chemokines With Critical COVID-19 Patients In-Hospital Mortality

**Figure 6:**
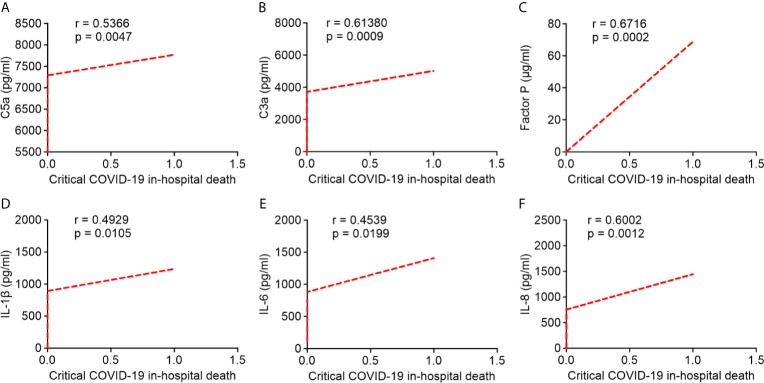
Correlation between serum complement components and inflammatory cytokine/chemokine levels in critical COVID-19 patients who died in hospital. **(A)** complement fragment C5a (*P* = 0.005); **(B)** complement fragment C3a (*P* < 0.001); **(C)** factor P (*P* < 0.001); **(D)** interleukin 1 β (IL-1β) (*P* = 0.01); **(E)** interleukin 6 (IL-6) (*P* = 0.02); **(F)** interleukin 8 (IL-8) (*P* = 0.001).

Pearson’s correlation analysis showed that serum levels of C5a, C3a, and factor P strongly correlated with critical COVID-19 in-hospital death (*r* = 0.5366; *r* = 0.6138; *r* = 0.6716, respectively; **Figures 6A–C**). Likewise, serum IL-1β, IL-6, and IL-8 levels were positively correlated with critical COVID-19 in-hospital death (*r* = 0.4929; *r* = 0.4539; *r* = 0.6002, respectively; **Figures 6D–F**). Our results indicated that the overactivation of the complement system and higher levels of pro-inflammatory cytokines/chemokines in critical COVID-19 patients were significantly correlated with critical COVID-19 in-hospital mortality.

### Pro-Inflammatory Cytokine/Chemokine Profiles in the Serum of Critical COVID-19 Patients and Their Correlation With Serum Concentrations of Complement Proteins

We further investigated any potential correlation between pro-inflammatory cytokines/chemokines and anaphylatoxins and showed that the levels of C3a positively correlated with the levels of IL-6 (*r* = 0.3886, *P* = 0.0498) and IL-1β (*r* = 0.3517, but *P* = 0.0781; **Table 2**). Additionally, C5a levels mildly, correlated with the levels of IL-1β (*r* = 0.2979, but *P* = 0.1394) and IL-6 (*r* = 0.2862, but *P* = 0.1564), however these results were not significant (**Table 2**). In addition, serum factor P levels positively correlated with the levels of C3a (r = 0.6766, *P* = 0.0001). We also observed a positive correlation between C5a and C3a levels (*r* = -0.5672, *P* = 0.05; **Table 2**).

**Table 2:** Correlation between serum complement proteins and inflammatory. cytokines/chemokines.

Variable	Pearson (r)	*p-*value
C3a	IL-6 (r = 0.3886)	0.0498
	IL1-β (r = 0.3517)	0.0781
C5a	IL-6 (r = 0.2862)	0.1564
	IL-1β (r = 0.2979)	0.1394
	C3a (r = -0.5672)	0.0500
Factor P	C3a (r = 0.6766)	0.0001

## Discussion

In this study, we sought to determine the level of complement factors and pro-inflammatory cytokines/chemokines in the critical and mild patient responses to SARS-CoV-2. This study offers the first insights into the protein expression profile of the complement factors during SARS-CoV-2 infection. Different markers of human serum obtained from patients with COVID-19 and healthy controls were analyzed. The concentration of complement inflammatory mediators (C3a and C5a), responsible for attracting phagocytic cells to infection sites, were higher in critical COVID-19 patients than in the mild and healthy groups. However, lower levels of complement regulators were associated with the critical groups. We also analyzed a panel of 12 inflammatory cytokines/chemokines, including IL1-α, IL1-β, IL-2, IL-4, IL-6, IL-8, IL-10, IL-12, IL-17A, IFN-γ, TNF-α, and GM-CSF, in the sera obtained from critical and mild patients diagnosed with SARS-CoV-2 infection. Among these cytokines, serum levels of IL1-β, IL-6, IL-8, and TNF-α were found to be significantly increased in the critical COVID-19 patients relative to mild patients. In addition, the RANTES serum levels were significantly elevated in mild COVID-19 patients relative to the critical cases.

The complement system is an important component of the innate immune response to pathogens. Activation of complements by viral infections induces acute and chronic inflammation, as well as intravascular coagulation and cell damage. Consequently, organ failure and death occur (23, 26). Indeed, complement activation has been implicated in the pathogenesis of MERS-CoV and SAR-CoV infections, which is similar to the current pandemic SARS-CoV-2 infection (27).

C5a and C3a are potent mediators that induce inflammatory reactions. C5a triggers the recruitment of neutrophils and monocytes followed by their accumulation and activation. In addition, it initiates mast cells activation and degradation while increasing the induction of cytokines and vascular permeability (28, 29). This observation was evident following SARS-CoV infection (30) as well as demonstrated *in vitro* and *in vivo* following pathogenic H5N1 influenza virus (31). Our work has demonstrated that the concentrations of the pro-inflammatory mediators, anaphaylatoxin C3a and C5a, were significantly increased in critical COVID-19 patients relative to mild and non-infected groups. In turn, these anaphylatoxins likely enhanced the release of pro-inflammatory cytokines and chemokines (IL-1, IL-8, IL-6 and TNF-a) from phagocytic cells and T cells, as previously reviewed (19). This indicates that the amount of C3 is this group is higher than that of healthy volunteers. Additionally, the non-survival group demonstrated a higher concentration of C3a, C5a, and factor P in comparison to the critical survival group. A significant correlation was observed between serum complement components and inflammatory cytokines/chemokines levels in patients with critical COVID-19 in-hospital mortality.

A recent study on SARS-CoV infections has shown that C3 activation exacerbates acute respiratory distress syndrome (ARDS) which is associated with SARS-CoV. Low lung infiltration of neutrophils, inflammatory monocytes, and decreased concentration of cytokines and chemokines in the lungs and serum have also been reported (32). A deficiency of C3 in these patients led to low production of C3a and C3b, a skewed immune response toward Th2 and induction of regulatory T cell development from CD4+ cells (33, 34).

Another study (35) has described the increased serum levels of C5a in critical cases of COVID-19 patients, which is consistent with our findings. A study conducted in Italy showed high induction levels of C5a and C5b-C9 in the plasma of a COVID-19 infected group (36). These molecules were observed in the serum and lung tissue of mice infected with MERS-CoV (37). However, in severe COVID-19 patients, only the deposition of C5b-9 was observed and was significantly elevated in pulmonary microvasculature (22). Patients hospitalized with COVID-19, who were admitted to ICU, had elevated levels of C3b on red blood cells peaking on day 7, relative to healthy non-infected COVID-19 donors (38). Collectively, MERS-CoV, SARS-CoV, and SARS-CoV-2 over-activate the complement system and contributes to dysregulation of the host immune response. High serum levels of complement protein C5a have also been reported in non-survivor H5N1 patients compared with survivors (24). Animal studies have further demonstrated that complement C3 knockout mice are protected from lung inflammation and respiratory failure as well as inflammatory cytokines (32). Furthermore, a MERS-CoV mouse model treated with a C5a receptor (C5aR, CD88) blocker reduced lung inflammation and decreased viral replication in the lungs (39).

Furthermore, other proteins mediators of complement, such as C3, C4, and C5b-9, as well as inflammatory cells were found to be significantly increased in alveolar epithelial cells of the non-survivors infected with COVID-19. A probable inhibitory effect was noticed in this study when patients were treated with anti-C5a monoclonal antibody (35), which may be a potential strategy against SARS-CoV-2 infection. In addition, strong deposition of C5b-9 in the kidney tissue of patients infected with SARS-CoV-2 has recently been reported, which caused tissue damage (40).

To the best of our knowledge, this is the first study to report significantly elevated levels of other complement components such as factor P, C1q, C2, and C3 in COVID-19 patients in comparison to healthy non-infected individuals. Moreover, factor P was higher in the critical group than in the mildly affected group. However, no difference was observed between the critical and mildly affected groups in terms of C1q. These mediators have important roles in enhancing complement activation during SARS-CoV-2 infection. Thus, COVID-19 may trigger activation of complement *via* different pathways, especially the classical and alternative pathways, causing an accumulation of opsonin molecules, such as C3b, in COVID-19 patients. Another study showed that SARS-CoV, MERS-CoV, and SARS-CoV-2 can activate the lectin pathway through interactions between N-protein and MASP-2 (35). Hence, we can conclude that 1) the lower C2 and C1q level in critical patients is believed to complement the depletion during activation; 2) regulatory mediators are upregulated; and 3) the time of sample collection may have impacted the results, as levels were increased during the early stage and gradually decreased during infection.

Several regulatory molecules, including C1-INH, C4-BP, decay-accelerating factor (DAF/CD55), factor H, and factor I, control complement activation (11, 19). This is the first report regarding SARS-CoV-2 infection, showing that both critical and mild cases of COVID-19 patients have elevated levels of factor I, factor H, and C4-BP compared to those in non-COVID-19 healthy individuals (**Figure 3**). However, the critical groups had a lower proportion of these regulatory proteins, except for factor H, in their serum compared to mild cases (**Figures 3A–C**). This might be attributed to the high concentration of anaphylatoxins, C3a, and C5a, among the critical groups resulting from complement activation.

The first published autopsy study of severe COVID-19 infection showed an association between activation of the alternative pathway and lectin pathway cascades and microvascular injury and thrombosis, suggesting complement-mediated thrombotic microvascular injury syndrome through alternative and lectin pathway activation (22). Accumulated evidence demonstrated an association between coagulopathy (pulmonary embolism or venous, arterial, and/or microvascular thrombosis) and SARS-CoV-2 infection (41). In fact, systemic microthrombi and multi-organ injury following SARS‐CoV-1, MERS‐CoV, SARS‐CoV‐2, H1N1, H5N1, and H7N9 influenza infections have been described (24, 42). Patients with SARS-CoV-2 infection are characterized by overactivation of the complement pathways (17, 43). Meanwhile, data from COVID-19 patients showed that inflammation and respiratory failure are associated with systemic complement activation (44). A recent study confirmed that severe COVID-19 cases have a higher level of circulating C5a and sC5b-9, signifying C5a blockade as a potential treatment strategy to control and reduce disease severity (36, 44). Numerous randomized controlled trials reported increased survival in severe cases of COVID-19 patients treated with anti-C5 therapy (27, 36, 45). Collectively, these results confirm that inhibition of an over-activated complement response significantly reduces COVID-19 disease severity.

We have also demonstrated a significant correlation between complement concentration and coagulation in patients infected with SARS-CoV-2, compared to mild cases and healthy controls. Moreover, patients with high complement proteins showed increased platelet counts. Several studies have shown that COVID-19 patients with excessive coagulant factors are more susceptible to increased thrombosis and deteriorating clinical outcomes (21). Thus, the lack of C3 in mouse models demonstrated a decrease in apparent defect of thrombus and activation of platelets (46). However, after treatment with different anticoagulants (Clexane & heparins), the level was decreased. The levels were significantly decreased in the same group that received Clexane but not heparin. Moreover, critical patients treated with hydroxyl chloroquine and antiviral drugs (ritonavir and lopinavir) showed inhibition of coagulation with no thrombosis, although antiviral drugs (remdesivir, favipiravir) alone had no significant effect.

The levels of cytokines and chemokines in patients with COVID-19 remains controversial (47). Moreover, ICU patients have elevated plasma levels of IL-2, IL-7, IL-10, GSCF, IP10, MCP1, MIP1A, and TNF-α compared to non-ICU patients (13). However, one study has reported no significant differences in serum levels of TNF-α, IL-1, IL-8, and IL-10 in mild, severe, and critical ICU patients (48). Similarly, we did not observe differences in IL-10 levels between critical and mild COVID-19 patients.

Elevated serum levels of pro-inflammatory cytokines, including IL1-β, IL6, IL-12, IFN-γ, IP10, and MCP1 are associated with lung inflammation and damage in patients with SARS (49). Additionally, MERS-CoV infection has been reported to induce high levels of pro-inflammatory cytokines, including TNF-α, IFN-γ, IL-15, and IL-17 (50). Consistent with previous findings (51, 52), we also observed that serum levels of IL1-β, IL-6, IL-8, and TNF-α, but not IL1-α, IFN-γ, IL-2, IL-4, IL-10, IL-12, or IL-17A were significantly elevated in ICU patients compared to non-ICU patients, suggesting a possible role for T-helper-1 cell responses during SARS-CoV-2 infection. Moreover, the increased levels of Th1 cytokines, including IL-1β, IL-6, and TNF-α, may be associated with the cytokine storm that is associated with disease severity. Nonetheless, SARS-CoV-2 infection was also shown to induce increased levels of Th-2 cytokines, such as IL-4 and IL-10, which can suppress inflammation (49).

Previous studies have reported that TNF-α exerts strong antiviral activity against swine, avian, and human influenza viruses (53). These cytokines are involved in the regulation of inflammatory processes and infectious diseases (54). It has further been reported that TNF-α serum levels are increased in COVID-19 patients with higher levels detected in severe cases (13, 51, 52). However, normal levels of TNF-α have been reported in severe COVID-19 patients (55). Previous studies have also reported that overproduction of TNF-α is associated with poor disease outcomes in MERS-CoV and SARS-CoV (50, 56), while administration of anti-TNF-α antibody (certolizumab) may have positive effects on COVID-19 patients (57). Additionally, although elevated levels of TNF-α and IFN-γ contribute to lung damage and high case fatality in COVID-19 patients, combination therapy of anti-TNF-α and anti-IFN-γ neutralizing antibodies effectively reduces inflammatory cell death, tissue damage, and mortality (58)

IL-1 is actively involved in inflammatory response to infection (59). Meanwhile, SARS-CoV-2 has been shown to affect the activation and maturation of IL-1β, which can sequentially activate IL-6 and TNF-α (60–62). IL-1β also contributes to the cytokine storm induced by SARS-CoV-2 (63). In fact, most COVID-19 patients with severe illness have increased levels of IL-1β, which is associated with ARDS (64); therefore, inhibition of IL-1β can mitigate development of the cytokine storm that causes death in COVID-19 patients (65). Additionally, IL-1 and IL-18 are secreted following inflammasome activation and have critical roles in the regulation of innate and adaptive immune responses (66). Severe SARS-CoV-2 infection engages inflammasome activation and pyroptosis associated with elevated levels of IL-1ß. However, therapy targeting IL-1ß has demonstrated beneficial outcomes in the prevention of SARS-CoV-2-induced cell death (67). Similarly, early blockade of the IL-1 receptor was found to be a promising therapeutic strategy against hyperinflammation, development of the cytokine storm, and respiratory failure in COVID-19 patients (68).

Furthermore, IL-6 levels are elevated in patients with COVID-19 and are associated with a poor prognosis (48, 69, 70), and are further increased in severe COVID-19 associated symptoms compared to mild patients, suggesting the importance of this cytokine as a marker to monitor disease progression and development (52, 55). Additionally, the IL-6 levels were higher in patients who died from COVID-19 than in the recovered individuals (71). Recently, it has been reported that increased levels of IL-6 enhance the inflammatory process and contribute to cytokine storm thereby worsening prognosis (72); however, targeting IL-6 receptors (IL-6r) with a specific monoclonal antibody (tocilizumab) has been shown to mediate an effective therapeutic option in COVID-19 patients who are at risk of developing cytokine storms (69).

In addition, IL-8 is a strong pro-inflammatory cytokine that plays an essential role in the activation and recruitment of neutrophil cells during inflammation (73); neutrophilia is more frequently observed in severe COVID-19 patients than in the mildly affected patients (14), suggesting that IL-8 participates in the pathophysiology of COVID-19. Similarly, in our study, high IL-8 levels were associated with increased numbers of neutrophils, particularly in the ICU patients relative to mild patients and healthy normal controls. IL-8 also contributes to the occurrence of ARDS and results in a cytokine storm linked to death in patients infected with SARS-CoV or MERS-CoV (74, 75). It has been established that pre-treatment with an anti-CXCL8 antibody prevents the development of severe lung injury (76); thus, targeting this cytokine or its receptor may offer an effective treatment option for COVID-19 patients.

RANTES (CCL5) is a strong leucocyte chemoattractant that can activate and induce migration of several immune cells, including T cells, natural killer cells, dendritic cells, monocytes, basophils, and eosinophils, to the site of inflammation (77, 78). It has been reported that, at the early stage of SARS-CoV-2 infection, increased serum levels of CCL5 were observed in patients with mild COVID-19 symptoms compared to severe patients, suggesting that CCL5 may protect against viral infection, where virus-specific CD8^+^ T cells respond to the removal of the virus before lung inflammation occurs (79). In addition, it has been shown that mild cases, not severe COVID-19 patients, are characterized by high expression of clonally expanded CD8^+^ T cells in the bronchoalveolar fluid, which suggests the potential mechanisms underlying pathogenesis and recovery in mild patients (80). Moreover, in agreement with these findings, we found that serum levels of RANTES were significantly elevated in mild patients and critical ICU patients compared to healthy normal controls. However, RANTES levels were significantly increased in the mildly affected patients compared to the critical ICU patients (81). Taken together, these results suggest that targeting RANTES, at least in the early stages of viral infection, may enhance viral clearance and prevent the spread of the virus to the lung and other parts of the body. In addition, this finding highlights the fact that RANTES might be important in antiviral responses in patients mildly affected with COVID-19 but also contributes to cytokine storm and mortality in severe cases.

The detection of antibodies during SARS-CoV-2 provides insights into the disease diagnosis and clinical course of COVID-19 infection. However, recent studies have indicated that COVID-19 patients with high levels of antibody responses, including the total antibodies, were associated with COVID-19 disease severity (9, 82). Likewise, increased B cell activation and proliferation in severe COVID-19 patients is associated with adverse outcomes (83, 84).

Additionally, a higher antibody response during several viral infections, including COVID-19 and MERS-CoV, was associated with antibody-dependent enhancement of immunopathological and robust inflammatory response (85, 86). In this study, high levels of IgM were detected in critical COVID-19 patients. Our findings suggest that high levels of SARS-CoV-2 IgM response could act as an early biomarker to differentiate between critical and mild COVID-19 patients. Furthermore, this result provides evidence of the possible antibody-dependent enhancement phenomenon during SARS-CoV-2 infection. In fact, markedly-increased humoral immunity has been linked to antibody-dependent enhancement, which was observed during SARS-CoV-1 infection (87). Therefore, we believe that high levels of IgM may play a critical role in the immunopathology and severity of COVID-19. In any case, further studies are required to confirm this finding.

The limitations of the current study can be summarized as follows: 1) Assessment of T-helper-1 and T-helper-2 cytokine expression in the sera of infected patients may not reflect the host response against SARS-CoV-2 in the airways. 2) COVID-19 patients were not tracked, and serum samples were collected once and, therefore, it is difficult to determine the kinetics of cytokine levels. Furthermore, future studies should examine the expression of cytokines in bronchoalveolar fluid samples from COVID-19 patients to achieve a better understanding of the host immune response to SARS-CoV-2 infection. In addition, conducting multiple sampling time points may help to determine the peak levels for each cytokine.

In conclusion, we showed an increase in the serum levels of cytokines, including IL1-β, IL-6, IL-8, and TNF-α in ICU patients relative to mild COVID-19, while RANTES serum levels were increased in the mild, not ICU patients. In addition, SARS-CoV-2 infection contributes to the potent activation of complement mediators including C5a, C3a, factor P, factor I, factor C4-BP, and factor H. Despite the advantage of complement activation and protection against COVID-19, there is a strong association in the pathogenesis of SARS-CoV-2, especially in tissue inflammation and coagulation. Thus, this outcome might have contributed to tissue damage and dysfunction, whether in circulation or other organs. There is an urgent need to identify an effective therapeutic strategy against COVID-19 by targeting and suppressing excessive mediators of complements in controlling SARS-CoV-2 infection, as well as pro-inflammatory cytokines (IL1-β, IL-6, and TNF-α) and chemokines (IL-8 and RANTES) that might be used as predictive markers to evaluate disease severity and, thus, can be targeted as treatment options in COVID-19 disease.

## Data Availability Statement

The raw data supporting the conclusions of this article will be made available by the authors, without undue reservation.

## Ethics Statement

The studies involving human participants were reviewed and approved by The Institutional Review Board of King Fahad Medical City (IRB register number 20‒193). The patients/participants provided their written informed consent to participate in this study.

## Author Contributions

WA contributed to study design, planning, and supervision. WA and BA secured funding. MH and BA performed the experiments and data analysis. FA, AZA, and AAA conducted sample collection. AA and ME contributed to clinical data collection and manuscript revision. WA, BA, AM, and MH wrote the manuscript. All authors contributed to the article and approved the submitted version.

## Funding

This work was supported by the Targeted Research Grant Program of King Abdulaziz City for Science and Technology (grant number: 5-20-01-675-0002).

## Conflict of Interest

The authors declare that the research was conducted in the absence of any commercial or financial relationships that could be construed as a potential conflict of interest.
